# Monte Carlo modeling of the Elekta Versa HD and patient dose calculation with EGSnrc/BEAMnrc

**DOI:** 10.1002/acm2.13715

**Published:** 2022-08-19

**Authors:** Holly M. (Parenica) Paschal, Christopher N. Kabat, Pavlos Papaconstadopoulos, Neil A. Kirby, Pamela A. Myers, Timothy D. Wagner, Sotirios Stathakis

**Affiliations:** ^1^ Department of Radiation Oncology, School of Medicine The University of Texas Health Science Center at San Antonio San Antonio Texas USA; ^2^ The Netherlands Cancer Institute Amsterdam The Netherlands

**Keywords:** dose calculation, Monte Carlo, quality assurance

## Abstract

**Introduction:**

Numerous studies have proven the Monte Carlo method to be an accurate means of dose calculation. Although there are several commercial Monte Carlo treatment planning systems (TPSs), some clinics may not have access to these resources. We present a method for routine, independent patient dose calculations from treatment plans generated in a commercial TPS with our own Monte Carlo model using free, open‐source software.

**Materials and methods:**

A model of the Elekta Versa HD linear accelerator was developed using the EGSnrc codes. A MATLAB script was created to take clinical patient plans and convert the DICOM RTP files into a format usable by EGSnrc. Ten patients’ treatment plans were exported from the Monaco TPS to be recalculated using EGSnrc. Treatment simulations were done in BEAMnrc, and doses were calculated using Source 21 in DOSXYZnrc. Results were compared to patient plans calculated in the Monaco TPS and evaluated in Verisoft with a gamma criterion of 3%/2 mm.

**Results:**

Our Monte Carlo model was validated within 1%/1‐mm accuracy of measured percent depth doses and profiles. Gamma passing rates ranged from 82.1% to 99.8%, with 7 out of 10 plans having a gamma pass rate over 95%. Lung and prostate patients showed the best agreement with doses calculated in Monaco. All statistical uncertainties in DOSXYZnrc were less than 3.0%.

**Conclusion:**

A Monte Carlo model for routine patient dose calculation was successfully developed and tested. This model allows users to directly recalculate DICOM RP files containing patients’ plans that have been exported from a commercial TPS.

## 1 INTRODUCTION

The goal of radiation therapy is to deliver a prescribed amount of dose to a tumor while simultaneously limiting the dose that normal tissues receive. Targets of cancerous tissues that do not receive an adequate dose of radiation are at risk of local recurrence or spread of the disease. The International Commission on Radiation Units and Measurements has stated that doses delivered to patients must be within 5% of the dose prescribed by the radiation oncologist.^1^ Studies have shown that a 5% deviation in dose can result in a change of the tumor control probability from 10% to 20%.^2^ Conversely, a 5% change in dose delivered can increase the normal tissue complication probability by up to 30%. With all the possible sources of error (e.g., dose calculation, patient setup, and data transfer), ensuring that the dose delivered to the patient is within 5% of the prescribed dose can be challenging, and each step in the radiation oncology workflow must be as accurate as possible.

Dose calculation is a common source of error and uncertainty. Even if all other aspects of the patient's treatment are flawless and the treatment plan generated appears satisfactory (i.e., the tumor appears to be receiving an adequate dose and the normal tissues are sufficiently spared), it is irrelevant if the dose calculation algorithm used by the treatment planning system (TPS) is inadequate. Dose calculation accuracy depends heavily on the quality of the simulation CT the patient undergoes before they begin treatment. High‐density objects, such as metal implants or dental filling materials, negatively impact the quality of the CT scan acquired, thus making organ delineation and dose calculation in the TPS difficult and lacking accuracy.^3^, ^4^, ^5^, ^6^, ^7^, ^8^, ^9^, ^10^, ^11^ Additionally, small radiation fields lack lateral electronic equilibrium, and this phenomenon can present challenges for accurate dose calculation.^11^, ^12^, ^13^, ^14^

Currently, redundant independent dose calculations or dose/monitor unit (MU) verification serves as a standard for treatment planning QA, complementing measurement‐based patient‐specific QA.^15^, ^16^ Intensity‐modulated radiation therapy plans are characterized by steep dose gradients and are significantly more complex than conventional 3D plans. This increased complexity further necessitates the need for independent dose or MU verification.^17^ The American Association of Physicists in Medicine Task Group 219 (TG‐219) outlines a variety of the commercially available solutions that exist for secondary dose verification and discusses the advantages and disadvantages of each method. Many commonly used dose and MU verification programs only verify dose or MU delivered to a single point and do not provide a 3D dose verification. A secondary TPS may be used for independent dose verification but must use independent beam data and/or utilize a different dose calculation algorithm.^17^

Many studies have shown that the Monte Carlo dose calculation method is the most accurate algorithm for calculating dose in these challenging scenarios.^5^, ^18^, ^19^, ^20^, ^21^, ^22^, ^23^, ^24^ At the time TG‐219 was published, there were no commercially available secondary MU verification systems that utilized the Monte Carlo method for dose calculation; however, some commercial systems utilize Monte Carlo for dose calculation in treatment planning.^17^ One example is the Elekta Monaco TPS. The beam model in Elekta's Monaco TPS is based on a virtual fluence model for photons and relies on the input of depth–dose curves, output factors, and lateral dose profiles.^25^, ^26^ Although this beam model has a speed increase from a full Monte Carlo simulation, it does not simulate the scatter radiation produced by MLCs. A true Monte Carlo model will directly simulate particle interactions with the full geometry of the linac. This knowledge, along with the results of previous studies, has motivated us to create an independent, full Monte Carlo model of the Elekta Versa HD linear accelerators (linacs) in our clinic.

Although the value of a commercially available Monte Carlo TPS is evident, there may be clinics without access to such a system. These places could benefit from free, open‐source software like the EGSnrc user codes. There have been previous groups to model Elekta's 160 leaf Agility collimator.^27^, ^28^, ^29^ However, to the best of our knowledge, there is currently no model of Elekta's Versa HD linear accelerator that can be used for routine dose calculation on patients' CT datasets. We have developed a method for routine, independent patient dose calculations from treatment plans generated in a commercial TPS with our own Monte Carlo model. Additionally, this study sought to detect any shortcomings or errors that exist within our model and methodology. We have evaluated patient plans and recalculated them in our Monte Carlo model and compared these to a well‐established commercial Monte Carlo TPS (i.e., Monaco). Our overall aim is to provide the framework for clinics to develop their own true Monte Carlo model for independent dose verification and second checks.

## 2 MATERIALS AND METHODS

### 2.1 Modeling the Elekta Versa HD linear accelerator in BEAMnrc

The modeling of the 6‐MV photon beams for the Elekta Versa HD linear accelerator was completed using the EGSnrc/BEAMnrc user codes.^30^, ^31^ The EGSnrc software was selected for its ease of use in modeling medical linear accelerators. All modeling and simulations were performed using the Windows 10 operating system, installed on an Intel Xeon Gold 6138 CPU with 96.0 GB of RAM, with 40 cores and 80 logical processors with a base speed of 2.00 GHz.

**FIGURE 1:**
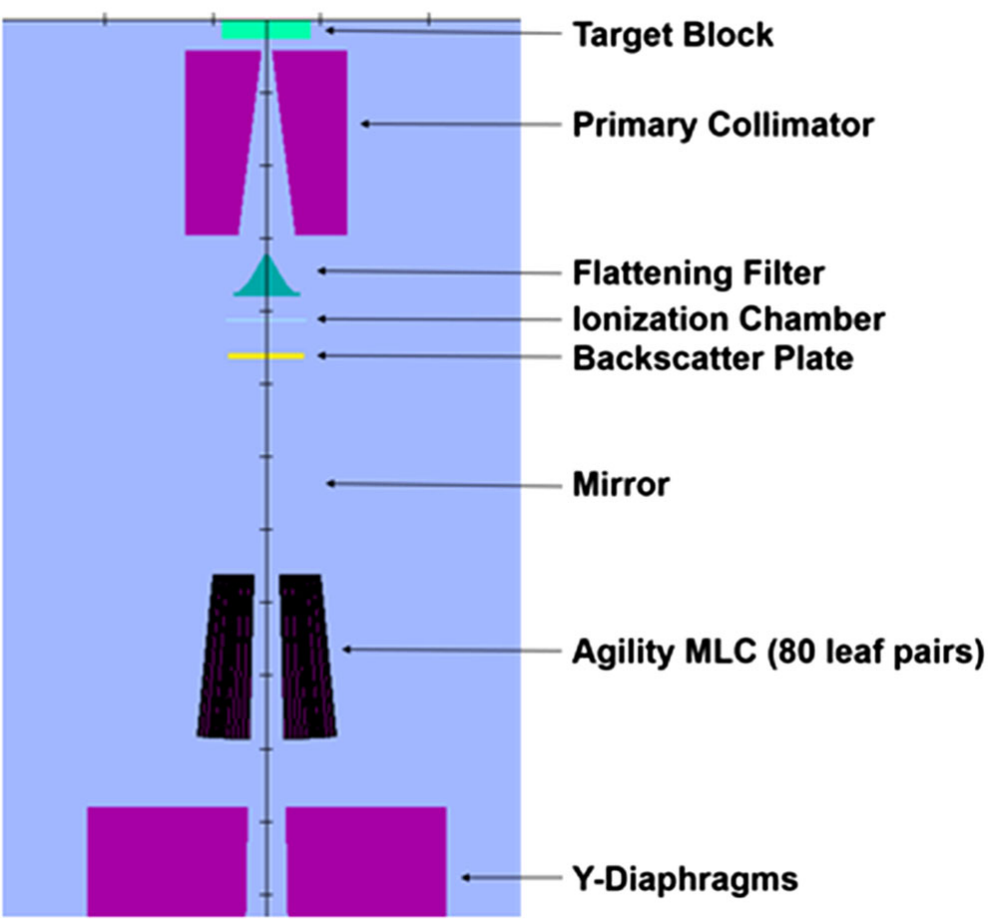
Preview of the component modules used to model the Elekta Versa HD. The preview of the accelerator shown is in the *Y*–*Z* plane, with *Z* being the direction of the beam.

All Monte Carlo modeling data was provided by Elekta (Stockholm, Sweden) and is proprietary information. Figure 1 shows the various component modules (CMs) in BEAMnrc representing the Elekta Versa HD. The initial source was simulated using ISOURC = 19 (elliptical beam with Gaussian distributions in *X* and *Y*), with the incident electron energy set to 6.4 MeV. All phase‐space files, generated in BEAMnrc, included a full simulation of the linear accelerator head. All simulations used an electron cutoff energy (ECUT) of 0.7 MeV and a photon cutoff energy (PCUT) of 0.01 MeV. Electron range rejection was set to 2 MeV. These values are commonly used and are well‐supported by literature [27, 28, 31]. The field sizes modeled ranged from 2 cm × 2 cm to 30 cm × 30 cm. All phase‐space files were scored at 100 cm from the target, behind the slab of air modeled in BEAMnrc. Two billion histories were used in each simulation for generating phase‐space data. All simulations were performed using parallel processing across 80 logical processors. Directional bremsstrahlung splitting (DBS) was implemented to aid in the reduction of simulation times.^31^

All dose calculations were performed using the EGSnrc/DOSXYZnrc user codes.^30^, ^32^ For each dose calculation, there were 9.0 × 10^9^ histories simulated to achieve a dose statistical uncertainty below 1%. Statistical uncertainty is defined as the average fractional uncertainty for the highest 50% of doses, and this value is reported by DOSXYZ at the completion of dose calculation. Because more histories were simulated in DOSXYZnrc than in BEAMnrc, the NRCYCL variable (i.e., the number of times to recycle each particle in the phase‐space source) was set to zero. By setting this variable equal to zero, DOSXYZ will automatically calculate an appropriate number of times to fully sample the phase‐space source and attempt to prevent it from being restarted.^32^ The global ECUT was set to 0.7 MeV and the global PCUT was set to 0.01 MeV. Range rejection (ESAVE) was set to 2.0 MeV.^27^, ^28^, ^31^ The percent depth–dose (PDD) curves and beam profiles obtained from dose calculation in DOSXYZ were compared to measurements made in water. All water tank measurements were made using the PTW BEAMSCAN water tank with the PTW Semiflex 31010 ion chamber (PTW, Freiburg) at an SSD of 100 cm for field sizes of 3 cm × 3 cm and larger. The PTW 60012 diode was used for field sizes of 2 cm × 2 cm and below. The detectors were daisy‐chained at 3 cm × 3 cm. Output factors were determined at 10‐cm depth in water and normalized to a 10 cm × 10 cm field for all field sizes.

### 2.2 Patient selection

Ten previously treated patients with a variety of treatment sites were randomly selected for dose recalculation using our in‐house Monte Carlo model, which utilized the BEAMnrc/DOSXYZnrc user codes.^30^, ^31^, ^32^ The patient cohort consisted of three head‐and‐neck cancer patients, three lung cancer patients, two gynecological cancer patients, and two prostate cancer patients. All patients were treated with 6‐MV VMAT photon beams, and each plan consisted of two arcs. Table 1 presents an overview of the patient cohort, including their assigned identifier. Each patient's plan was created in the Philips Pinnacle TPS, and the dose was initially calculated using the Collapsed Cone Convolution Superposition algorithm. The DICOM RP and DICOM RS (DICOM Version 3) files were exported to Elekta's Monaco TPS, and the dose was recalculated using the Monte Carlo algorithm in Monaco, with the dose‐to‐medium reported. All calculations in Pinnacle and Monaco were done with a 0.3 cm × 0.3 cm × 0.3 cm dose grid resolution. Doses calculated with Monaco's Monte Carlo algorithm were to be used for comparison with our in‐house Monte Carlo algorithm. Both the Pinnacle TPS and Monaco TPS have been previously verified for accuracy.^33^, ^34^ Patients’ DICOM RP files (files containing plan information), RD files (files containing dose information), and CT datasets were exported from the Pinnacle TPS.

**TABLE 1:** Summary of patient cohort and the number of histories simulated for each patient

	Treatment site	No. of fractions	Prescription dose (cGy)	PTV volume(s) (cc)	Number of control points per arc	Number of histories simulated in DOSXYZnrc
Patient 1*	Head and neck	33	6996/6000/5700	498.6/835.9/234.1	180	3.0 × 10^8^
Patient 2	Head and neck	17	3060	235.4	108	2.5 × 10^8^
Patient 3*	Head and neck	33	6996/6000	391.4/934.6	180	4.0 × 10^8^
Patient 4*	Lung	30	6600/6000	35.3/234.2	122	4.0 × 10^8^
Patient 5*	Lung	33	6600/6000	146.6/509.2	112	4.0 × 10^8^
Patient 6	Lung	30	6000	83.2	118	2.5 × 10^8^
Patient 7	Prostate	39	7800	98.1	178	4.0 × 10^8^
Patient 8	Prostate	30	7200	137.7	142	4.0 × 10^8^
Patient 9*	Pelvis (gynecological)	25	5750/5000/4500	42.8/74.1/2063.3	180	5.0 × 10^8^
Patient 10	Pelvis (gynecological)	25	2500	1446.2	180	4.0 × 10^8^

*Note*: Patients marked with * were prescribed a SIB.

Abbreviation: SIB, simultaneous integrated boost.

### 2.3 Patient treatment plan simulation with BEAMnrc

**FIGURE 2:**
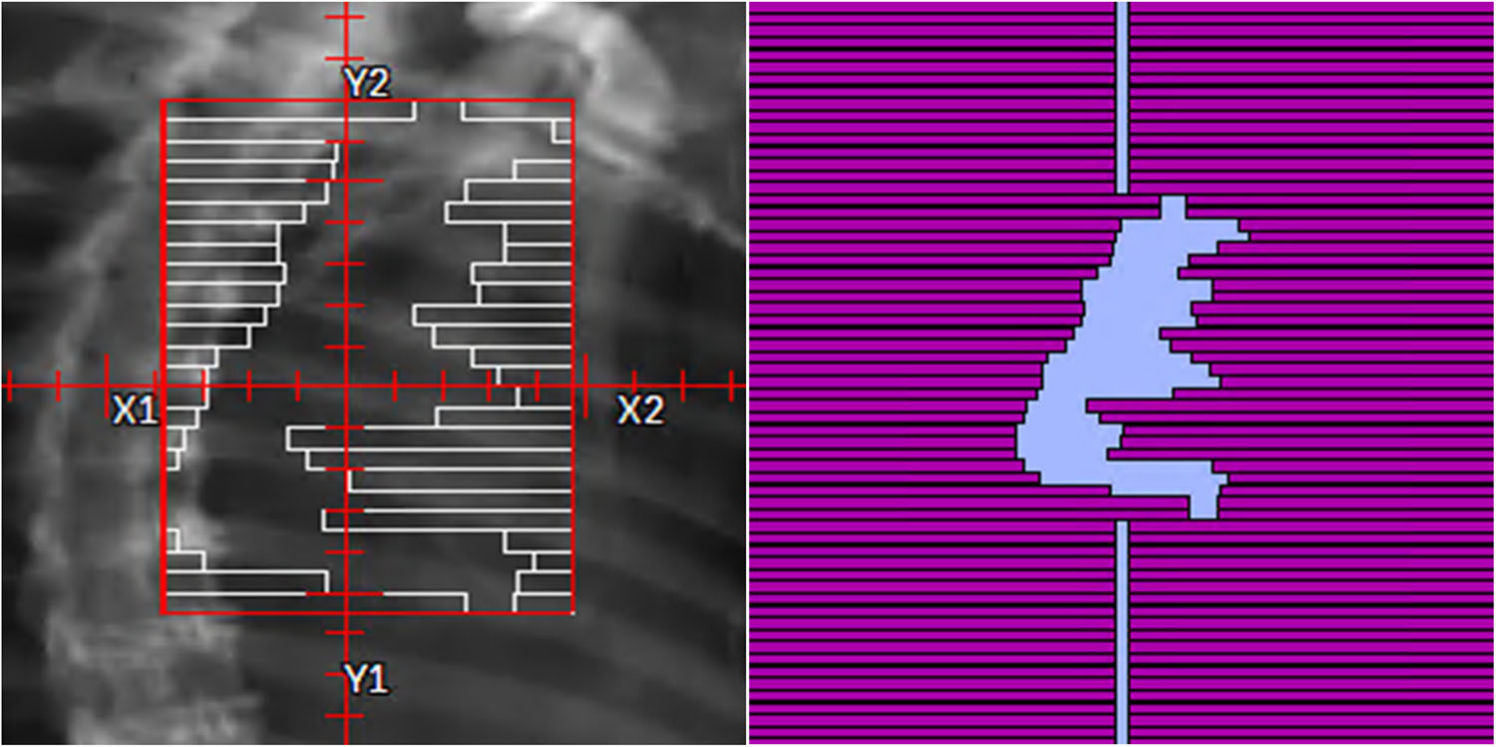
Beam's eye view preview. The beam's eye view of the MLCs of the first control point for a lung patient in both the Pinnacle treatment planning system (TPS) (left) and the BEAMnrc preview (right)

The BEAMnrc model of the Elekta Versa HD discussed previously was used for simulating all patient plans, including the initial source parameters (ISOURC = 19). One feature that particularly sets EGSnrc apart from other Monte Carlo codes is the ability to model the time‐synchronized components of a linear accelerator, namely, the MLCs, jaws, collimator, and gantry.^30^, ^31^, ^32^, ^35^ The SYNCMLCE and SYNCJAWS CMs were used to model the MLC and Y‐jaws, respectively. These CMs include the option to simulate the motion of individual leaves or jaws with time and can operate in “dynamic” mode (i.e., components are moving while the beam is on) or “step‐and‐shoot” mode (i.e., components are moving while the beam is off). All patients selected for dose recalculation were treated with 6‐MV VMAT photon beams; therefore, the “dynamic” setting was selected for each patient's simulation. To simulate leaf and jaw motion with respect to time, BEAMnrc requires the user to specify a *.sequence* file that contains all leaf/jaw motion data. The RP files exported from a TPS that contain all the plan information (including leaf/jaw positions) are exported in the DICOM format and cannot be used by EGSnrc. To address the issue of converting DICOM information from RP files into the *.sequence* file format required by BEAMnrc, a script was created in MATLAB that parses through the patient's DICOM RP file and extracts the information required by BEAMnrc, such as the leaf and jaw positions and the number of (MU) for each control point. The number of MUs dedicated to each control point is divided by the total number of MUs across all beams in the plan to represent the fraction of the total simulation that the specified geometry is present. Once the RP file is run through the MATLAB function, two *.sequence* files are produced (one for the MLC motion and the other for the Y‐jaws motion). Once the *.sequence* files are selected in the BEAMnrc GUI, the user can view the MLC and Jaw position for the first control point to verify the files are properly read into BEAMnrc. Figure 2 provides an example of this and displays the beam's eye view of the MLCs of the first control point for a lung patient in both Pinnacle and the BEAMnrc preview.

### 2.4 Patient dose calculation with DOSXYZnrc

All dose calculations were performed using the EGSnrc/DOSXYZnrc user codes. It was determined that reasonable statistical uncertainty (i.e., less than 3%) could be achieved using 2.5 × 10^8^–5.0 × 10^8^ histories in DOSXYZnrc when the “NRCYCL” feature was used. To simulate complex treatment geometries, such as VMAT, “ISOURC = 21”, or “Dynamic BEAM simulation source with multiple variable geometry settings” was selected. This setting uses a dynamic shared library between BEAM and DOSXYZ. Particle transport is simulated in the accelerator model in BEAM until the scoring plane is reached. From there, the simulation continues in DOSXYZ. This allows a full simulation of the accelerator head from the target to the scoring plane for each control point.^35^ The MATLAB function described in the previous section will produce, in addition to the *.sequence* files, a text file that contains the relevant geometry information with time (i.e., isocenter position, gantry angle, collimator angle, and distance from the source) that DOSXYZnrc requires to simulate the dose deposition.

In EGSnrc, users have the option to convert CT datasets into the required *.egsphant* format required for dose calculation in DOSXYZnrc by using ctcreate.^32^, ^35^ This function allows the user to simulate dose deposition in anthropomorphic phantoms from CT data in the DICOM format. The user specifies the CT number upper bound, the mass material density lower and upper bounds, and the material corresponding to these parameters. The format of the resulting “.egsphant” file is rectilinear voxels in a plain text file containing the material and density data. For this study, all EGS phantoms were created with 0.3 cm × 0.3 cm × 0.3 cm voxels to match the dose grid resolution set in the TPS and the CT ramp specific to our clinic's CT scanner was utilized.

### 2.5 Dose calculation verification with Monaco

To easily compare the doses calculated in DOSXYZnrc with the Monaco TPS, the PTW Verisoft (PTW, Freiburg) software was used. To compare two 3D dose distributions from calculated doses in Verisoft, both datasets must be in the DICOM format. Because DOSXYZnrc produces dose files in the “.3ddose” format, the resulting dose files were converted into the DICOM format using a MATLAB script written by Mark Geurts that was obtained from the GitHub repository.^36^ The dose distributions for each patient calculated in DOSXYZnrc were compared to those calculated in Monaco (which served as the reference dose), and a 3D gamma score was obtained for each patient, using a criterion of 3%/2 mm (dose difference and distance‐to‐agreement, respectively), with global normalization and a 10% dose threshold.^37^

## 3 RESULTS

### 3.1 Initial modeling—PDDs, profiles, and output factors

**FIGURE 3:**
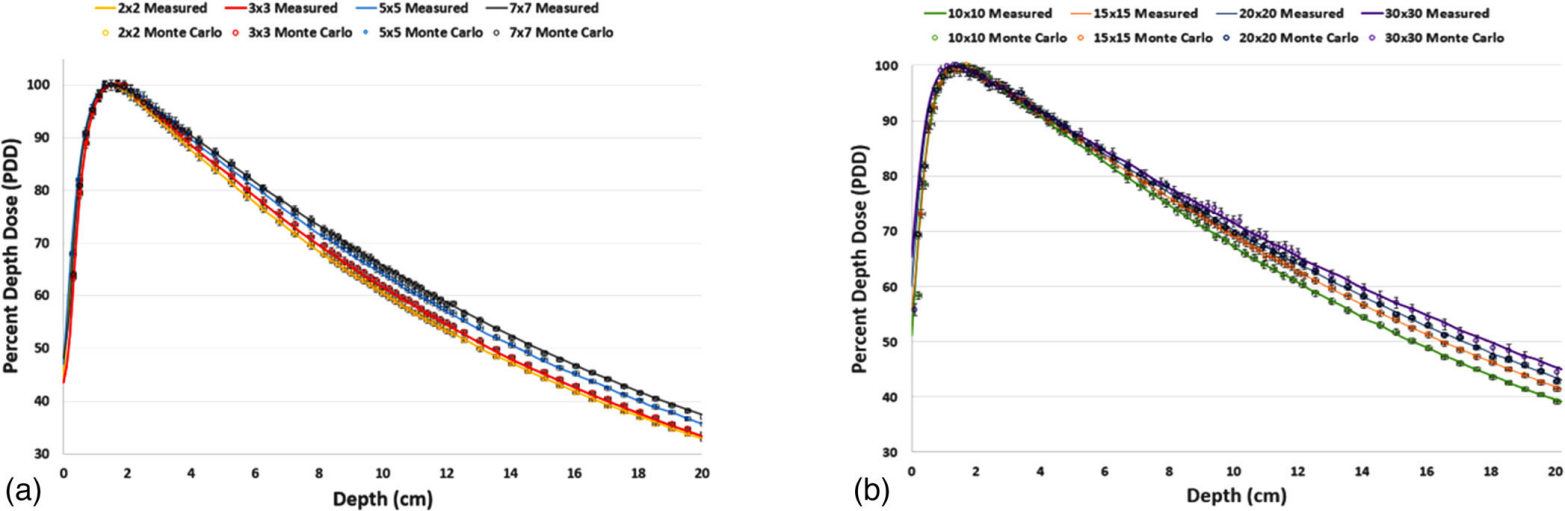
Percent depth–dose curves. Percent depth–dose curves of the small fields (a) and large fields (b) both measured and simulated in DOSXYZnrc. All *X* error bars shown are 1 mm and all *Y* error bars shown are 1%.

All dose calculations in DOSXYZnrc had less than 1% statistical uncertainty. The initial source was found to have a FWHM of 0.11 cm in both the *X* and *Y* directions, with an incident electron energy of 6.4 MeV. Figure 3a displays the PDD curves from the Monte Carlo simulations and water tank measurements of the small fields (i.e., 7 cm × 7 cm and smaller). Likewise, Figure 3b displays the PDD curves from the Monte Carlo simulations and water tank measurements of the large fields (i.e., 10 cm × 10 cm and larger). All error bars shown in the *X* direction represent a 1‐mm agreement and all error bars in the *Y* direction represent a 1% agreement, respectively. All field sizes simulated agreed with measured data within 1% and 1 mm at all depths, meaning that the initial energy of electrons incident on the target (i.e., 6.4 MeV) in BEAMnrc was appropriately selected.

**FIGURE 4:**
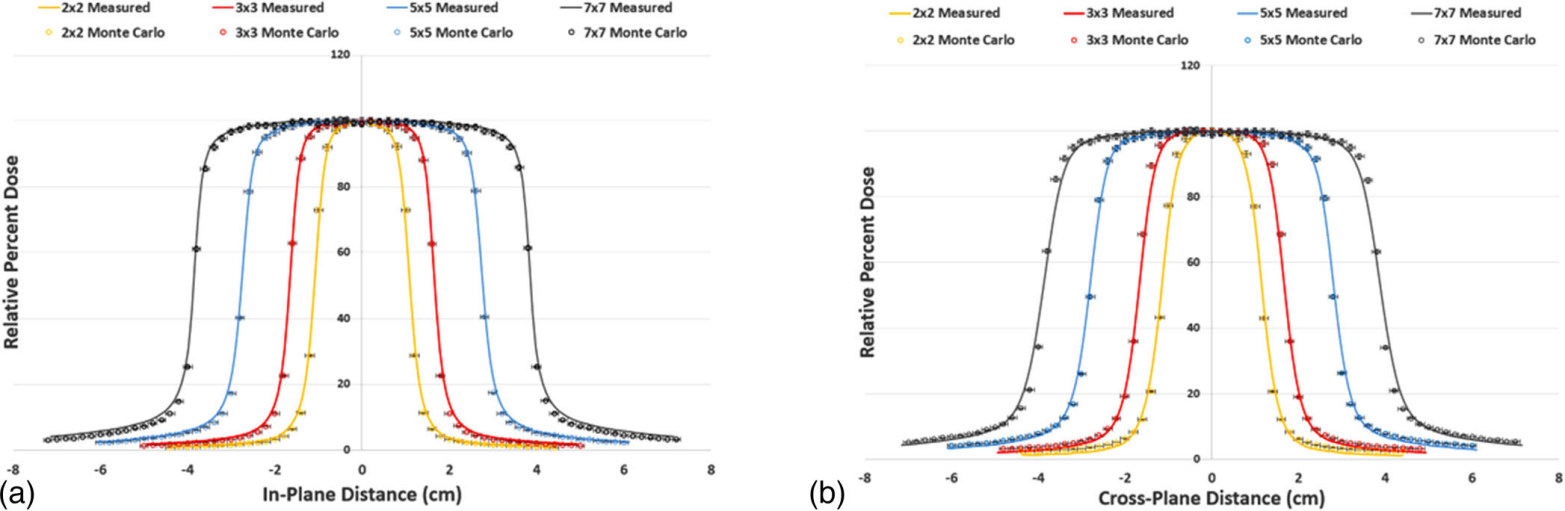
Small field profiles, in‐plane and cross‐plane. Beam profiles of the small fields, both measured and simulated, for the fields defined by the jaws (a) and fields defined by the MLCs (b). All *X* error bars shown are 1 mm and all *Y* error bars shown are 1%.

Figure 4a displays the in‐plane beam profiles of the small fields (i.e., 7 cm × 7 cm, 5 cm × 5 cm, 3 cm × 3 cm, and 2 cm × 2 cm), both measured and simulated, at 10‐cm depth. The in‐plane direction is the *Y* direction in BEAMnrc and is defined by the jaws. Likewise, Figure 4b displays the cross‐plane beam profiles of the small fields, both measured and simulated, at 10‐cm depth. The cross‐plane direction is the *X* direction in BEAMnrc and is defined by the MLC. All error bars shown in the *X* direction represent a 1‐mm agreement and all error bars in the *Y* direction represent a 1% agreement, respectively. All field sizes simulated showed good agreement with measured data, within 1% and 1 mm, particularly in the penumbra region of the profile (i.e., 80%–20% of the maximum dose at 10‐cm depth).

**FIGURE 5:**
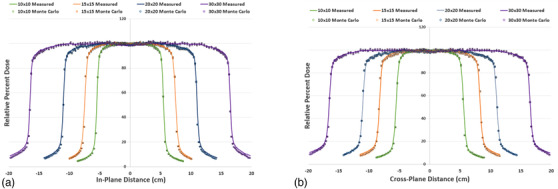
Large field profiles, in‐plane and cross‐plane. Beam profiles of the large fields, both measured and simulated, for the fields defined by the jaws (a) and fields defined by the MLCs (b). All *X* error bars shown are 1 mm and all *Y* error bars shown are 1%.

Figure 5a displays the in‐plane beam profiles (i.e., defined by the jaws) of the large fields (i.e., 10 cm × 10 cm and larger), both measured and simulated, at 10‐cm depth. Figure 5b displays the cross‐plane beam profiles (i.e., defined by the MLC) of the large fields, both measured and simulated, at 10‐cm depth. All error bars shown in the *X* direction represent a 1‐mm agreement and all error bars in the *Y* direction represent a 1% agreement, respectively. All field sizes simulated showed good agreement with measured data, particularly in the penumbra region of the beam profiles. Regions below 20% of the maximum dose at 10‐cm depth showed the greatest discrepancy from the measured data, particularly for the 30 cm × 30 cm field. However, the Monte Carlo simulations were still within reasonable agreement with measured data for these low‐dose regions.

**FIGURE 6:**
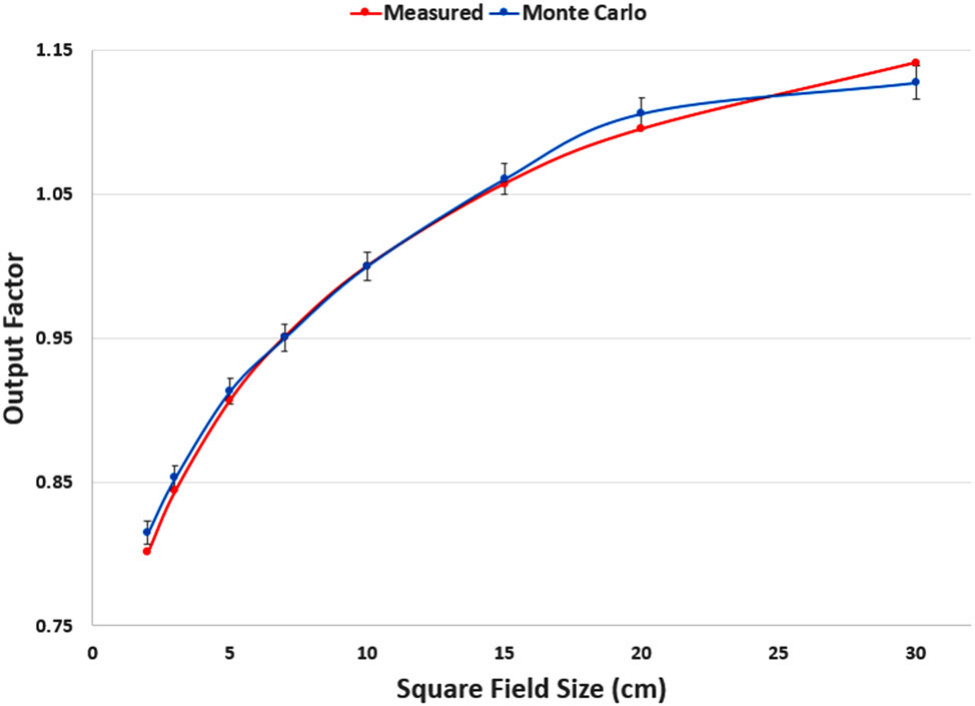
Output factors. Output factors for all field sizes, both measured and simulated. All *Y* error bars shown are 1%.

Figure 6 displays the output factors, both measured and simulated, for all field sizes. The *Y* error bars shown represent a 1% agreement. All simulated field sizes agreed with the measured data within 1%.

### 3.2 Patient dose calculations

**FIGURE 7:**
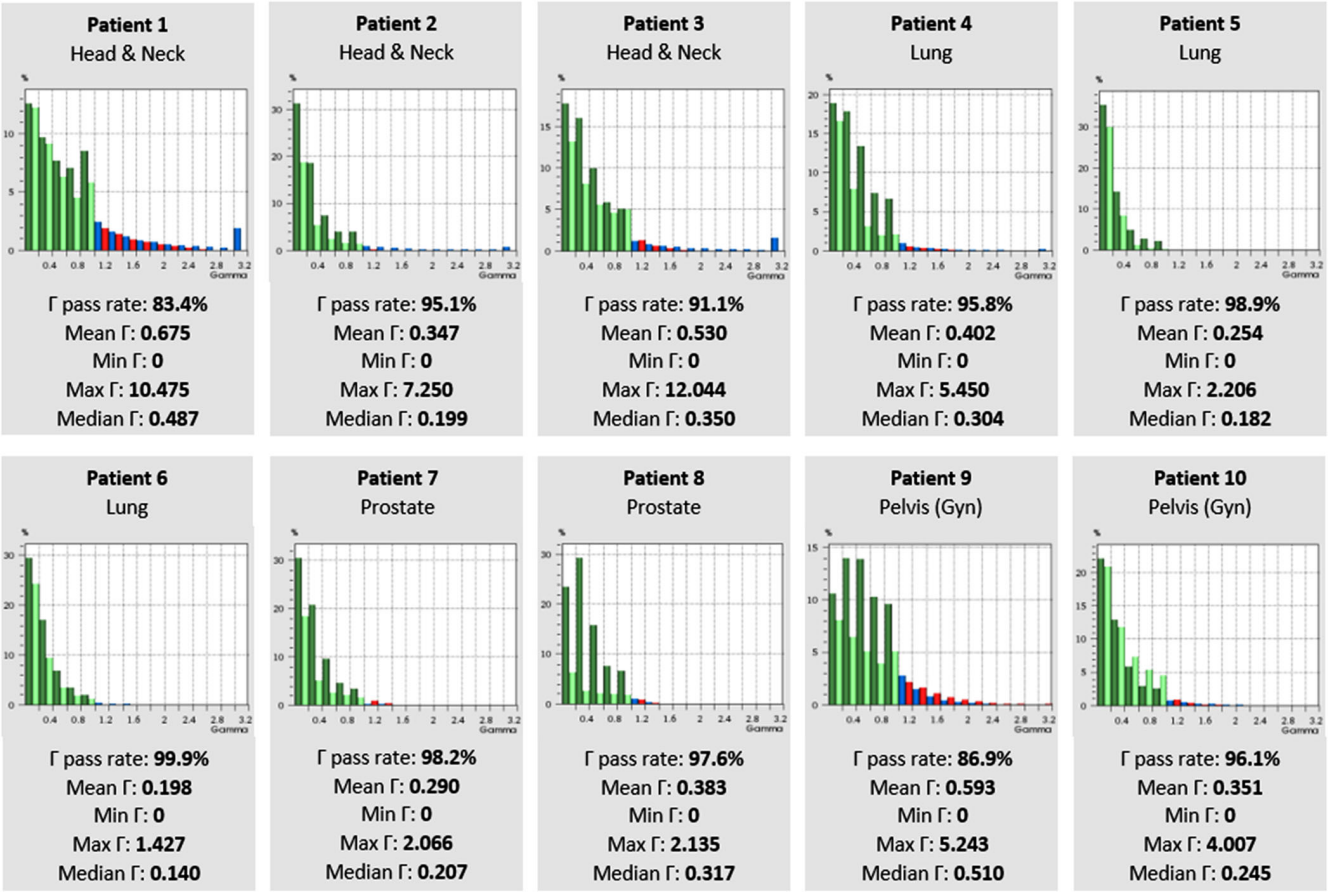
Gamma passing rate histograms when compared to Monaco. All patients’ gamma index histograms and relevant gamma statistics for plans recalculated in DOSXYZnrc compared to Monaco

Table 2 displays the results of each patient's 3D gamma when doses calculated in DOSXYZnrc were compared to doses calculated in the Monaco TPS. The statistical uncertainty for each patient's dose from DOSXYZnrc is also presented. Gamma passing rates ranged from 82.1% to 99.8%. Overall, lung and prostate patients showed the best agreement with doses calculated in Monaco. All statistical uncertainties in DOSXYZnrc were less than 3.0%, and patients with the largest field sizes generally showed the highest statistical uncertainty (e.g., Patients 1, 9, and 10). Figure 7 displays all patients’ gamma index histograms for patient plans recalculated in DOSXYZnrc when compared to Monaco, along with relevant 3D gamma statistics.

**TABLE 2:** Results of 3D gamma passing rates and statistical uncertainty in DOSXYZnrc

	Γ Pass rate (%)	Statistical uncertainty (%)
Patient 1 Head and neck	82.1	2.19
Patient 2 Head and neck	95.1	1.57
Patient 3 Head and neck	91.1	1.79
Patient 4 Lung	95.7	1.21
Patient 5 Lung	98.9	1.45
Patient 6 Lung	99.8	1.26
Patient 7 Prostate	98.2	0.97
Patient 8 Prostate	98.1	1.06
Patient 9 Pelvis (Gyn)	86.9	2.48
Patient 10 Pelvis (Gyn)	96.1	2.06

## 4 DISCUSSION

Many studies have demonstrated the superior accuracy of the Monte Carlo dose calculation algorithm when compared to other dose calculation methods, particularly in regions of inhomogeneity. The results of these studies motivated the development of a Monte Carlo model of the Elekta Versa HD for independent dose verification. Until more recently, the Monte Carlo dose calculation method was not used routinely in the clinic; however, advances in computing technology have made this calculation a possibility.

The use of a variance reduction technique such as DBS can have a profound impact on the time required for a Monte Carlo simulation to complete. Initially, DBS was not implemented during initial testing, and only uniform bremsstrahlung splitting was used. These initial simulations would take 4 h for 8.0 × 10^7^ histories in DOSXYZnrc, and the resulting statistical uncertainties were unacceptable (typically around 3.5%). However, by implementing DBS, 2.5–5.0 × 10^8^ histories could be simulated in DOSXYZnrc with statistical uncertainties of less than 3.0%. These simulations were completed in under 2 h, with many finishing in under an hour and a half. A greater efficiency gain is likely possible with further optimization of the DBS algorithm, namely, the optimization of the positions of the Russian roulette and electron splitting planes.^31^, ^38^ Additional speed improvements are necessary for this model to be used effectively in a clinical setting.

**FIGURE 8:**
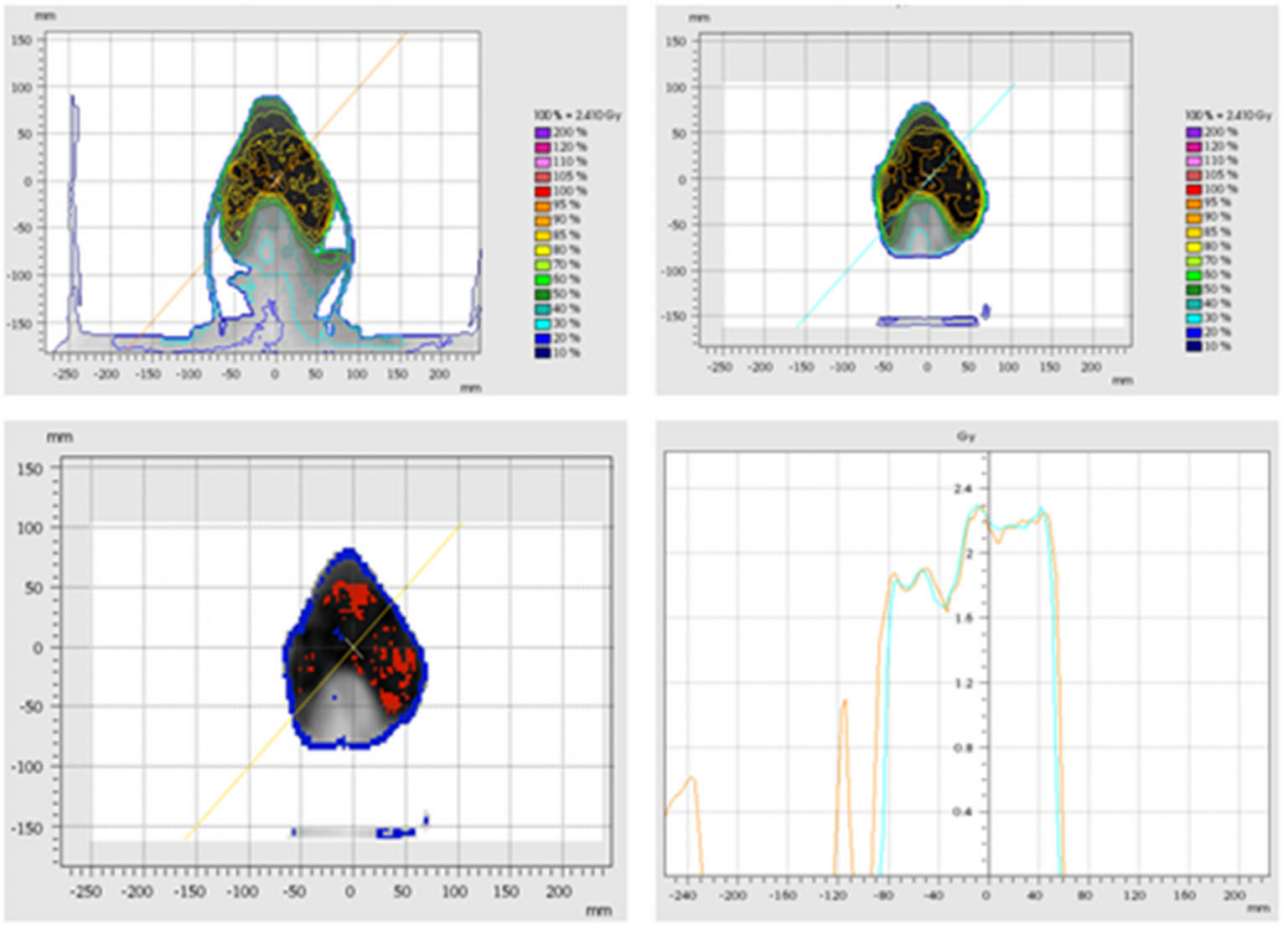
Head‐and‐neck patient single slice results (axial). Isodose lines for Patient 1 (head‐and‐neck) from DOSXYZnrc (top left) and Monaco (top right) for a single axial slice. The bottom left figure represents the “failed points” of a 2D gamma analysis. Voxels in red indicate that the dose calculated in Monaco was higher than the dose calculated in EGSnrc, whereas voxels in blue indicate that the dose calculated in Monaco was lower than the dose calculated in DOSXYZnrc. The bottom right figure displays the absolute dose profile of the positive diagonal slope seen in orange for EGSnrc and light blue for Monaco.

**FIGURE 9:**
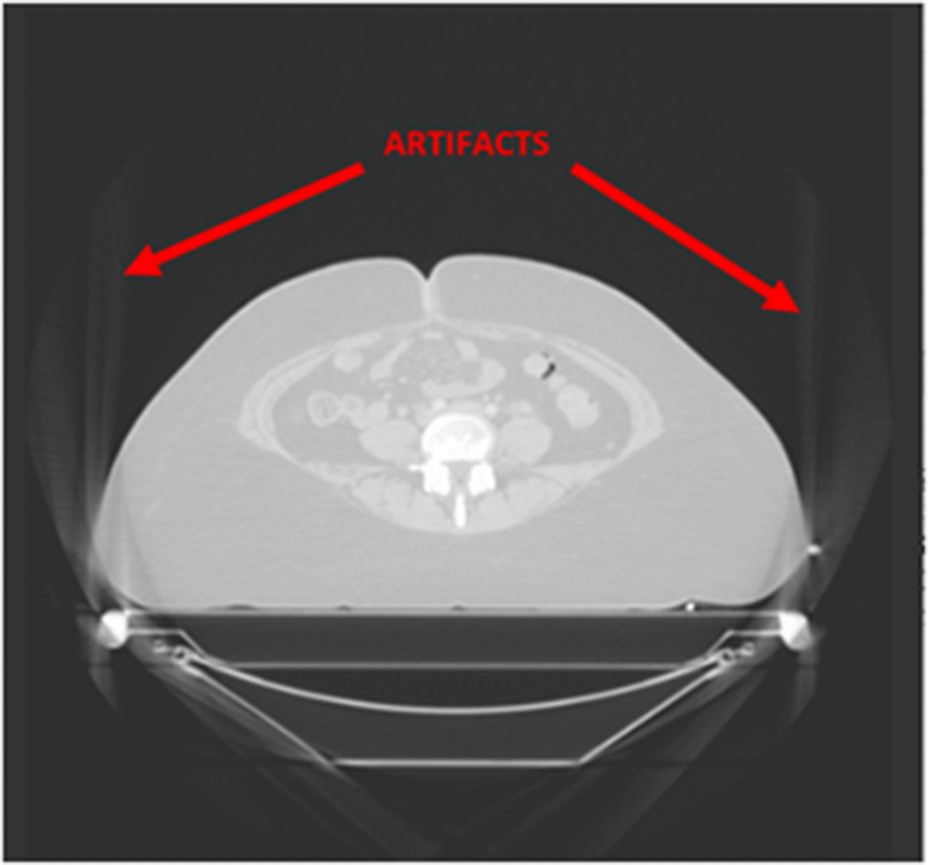
Artifacts in gynecological patient CT. Artifacts in Patient 9′s CT data. The presence of artifacts can lead to inaccuracies in the EGS phantom, which can lead to inaccuracies in dose calculation.

The Monte Carlo model presented in this study generally shows good agreement when compared to the Elekta Monaco TPS, a commercial Monte Carlo–based TPS. Although it will take a larger patient cohort to be able to conclude the accuracy of this Monte Carlo model with respect to anatomical site, this study suggests that head‐and‐neck cancer patients could be more susceptible to dose discrepancies when compared to a commercial TPS. Patient 1, a head‐and‐neck patient, had the lowest 3D gamma score of all the patients in this study. Figure 8 shows that DOSXYZnrc calculated a higher dose on the patient's skin surface but doses at deeper depths were lower than what Monaco calculated. This is likely due to the immobilization equipment (i.e., the facemask and Vac‐Lok bag) that was included in the raw CT data used to create the EGS phantom for dose calculation in DOSXYZnrc. In commercial TPS, the user can define the external contour of the patient, and voxels outside of this contour are considered air and are not included in the dose calculation (except for the table). Although the CT ramp specific to our machine was used to create EGS phantoms for dose calculation, no feature allows the user to explicitly define the patient's external boundary without changing the CT ramp to incorrect values. For example, in a commercial TPS, if the bag used for immobilization has a density of 0.1 g/cm^3^, the user can establish a density threshold of a higher value so that the bag is not included in the patient's dose calculation. In ctcreate, assigning voxels as air that are outside of the patient will cause voxels within the patient with the same density/CT number to also be assigned as air when they may be low‐density tissue (like lung). Patients with a treatment volume surrounded by large amounts of immobilization equipment, such as Patient 1, were susceptible to doses being lower at larger depths when compared with Monaco. Although other patients could have much of their immobilization equipment excluded from the phantom creation (i.e., cropped out) in ctcreate, this was not possible for Patient 1 due to the location of their PTV. Patient 9′s CT data suffered from artifacts due to the limited field of view (FOV), as seen in Figure 9. These artifacts could not be cropped out of the EGS phantom without also cropping out part of the patient within the treatment field and likely contributed to the loss of particles reaching the patient's surface because they were assigned the incorrect density in ctcreate. Similarly, Patient 1 also suffered from CT artifacts that were included in the dose calculation (shown in the top left of Figure 8), which likely further impacted this patient's dose calculation accuracy. In a commercial TPS, the planner would have the ability to contour these artifacts and assign them the correct density (i.e., air) and synthetically reconstruct the regions of the patient that are outside the FOV, but this solution is not easily obtained in ctcreate and DOSXYZnrc. This caused DOSXYZnrc to calculate a lower dose in some regions of the patient and contribute to this patient's poor 3D gamma score.

Another limitation of ctcreate is the feasibility of including treatment couch structures in the EGS phantom. The couch used during simulation is different from the couch used during treatment and, in a commercial TPS, users can easily create a structure of the treatment couch to be included in the patient's dose calculation. For this study, the couch structures were not included in the range of CT data to be included in the EGS phantom. Likewise, the couch structures in Monaco were overridden to the density of air and were not included in the dose calculations. This allowed us to compare the doses calculated for the patient in DOSXYZnrc and Monaco without the presence of the couch. Additional testing is necessary to accurately model the couch for a more realistic scenario of the patient's treatment.

Despite the inaccuracies resulting from discrepancies in ctcreate for some patients, 7 of the 10 patients in this study had 3D gamma passing rates above 95% and showed excellent agreement with Monaco when profiles of the absolute doses were studied. The lung and prostate patients showed excellent agreement with Monaco and had lower statistical uncertainties, likely because their treatment fields were smaller. Although this study did not specifically investigate the impact of small fields seen in stereotactic radiosurgery (SRS) or stereotactic body radiotherapy (SBRT), there were several patients with small targets (i.e., PTVs less than 100 cm^3^), including those prescribed a simultaneous integrated boost. Overall, patients with small targets still showed excellent agreement with Monaco. Simulating SRS/SBRT would give additional confidence in the MLC modeling and is an area for future investigation.

Overall, the in‐house Monte Carlo model developed in this study shows promising results when compared with a commercial Monte Carlo‐based TPS. Although a commercial Monte Carlo‐based TPS, like Monaco, is useful for independent dose calculation of the primary TPS, not all clinics have access to these capabilities. An in‐house model created with free, open‐source software (like EGSnrc) is a powerful tool that allows users the ability to develop their own second checks for dose verification. Additionally, an accurate Monte Carlo model can be useful for other research use within an institution.

## 5 CONCLUSIONS

The EGSnrc user codes, namely, BEAMnrc and DOSXYZnrc, were selected to create the Monte Carlo model for their relative ease of use in medical linear accelerator modeling. The Monte Carlo model was matched to PDD curves, beam profiles, and output factor measurements made with water tank measurements, and all were within 1% and 1‐mm agreement. For initial square field simulations, all statistical uncertainties were less than 1%.

The development and testing of the Monte Carlo model for routine patient dose calculation were described. This model allows users to directly recalculate DICOM RP files containing patients’ plans that have been exported from a commercial TPS (i.e., Pinnacle). The limitations that exist within the ctcreate software that creates EGS phantoms for dose calculation in DOSXYZnrc from patient CT data were also described.

The motivation for creating an independent Monte Carlo model stems from the results of our previous work and the work of many others: many commercial dose calculation algorithms are lacking in accuracy and require independent dose verification. Overall, the model described shows promise as an in‐house Monte Carlo system that can be utilized for patient dose verification and further research.

## CONFLICT OF INTEREST

The work described was supported by the National Center for Advancing Translational Sciences, National Institutes of Health (Grant no. TL1 TR002647). The content is solely the responsibility of the authors and does not necessarily represent the official views of the NIH. This work was also supported by the Cancer Prevention and Research Institute of Texas (CPRIT) Research Training Award (RP170345).

## AUTHOR CONTRIBUTIONS

The authors confirm contribution to the paper as follows: Study Conception and Design: Holly M. (Parenica) Paschal, Sotirios Stathakis; Data Collection: Holly M. (Parenica) Paschal, Christopher N. Kabat; Analysis and Interpretation of Results: Holly M. (Parenica) Paschal, Pavlos Papaconstadopoulos, Neil A. Kirby, Pamela A. Myers, Timothy D. Wagner, Sotirios Stathakis; Draft Manuscript Preparation: Holly M. (Parenica) Paschal, Sotirios Stathakis. All authors reviewed the results and approved the final version of the manuscript.
